# Child externalising and internalising behaviour and parental wellbeing during the Covid-19 pandemic

**DOI:** 10.14324/111.444/ucloe.000040

**Published:** 2022-09-16

**Authors:** Jill Portnoy, AnaCristina Bedoya, Keri Ka-Yee Wong

**Affiliations:** 1 School of Criminology and Justice Studies, University of Massachusetts Lowell, Lowell, MA, USA; 2 Department of Psychology & Human Development, University College London, UK

**Keywords:** Covid-19, parental stress, relational conflict, mental wellbeing, internalising problems, externalising problems, family, children, health

## Abstract

In this study we surveyed families’ experiences with parental depression, stress, relationship conflict and child behavioural issues during 6 months of the coronavirus (Covid-19) pandemic through the Covid-19: Global Social Trust and Mental Health Study. The current analyses used data collected from online surveys completed by adults in 66 countries from 17 April 2020 to 13 July 2020 (Wave I), followed by surveys 6 months later at Wave II (17 October 2020–31 January 2021). Analyses were limited to 175 adult parents who reported living with at least one child under 18 years old at Wave I. Parents reported on children’s level of externalising and internalising behaviour at Wave I. At Wave II, parents completed self-reported measures of stress, depression and inter-partner conflict. Child externalising behaviour at Wave I significantly predicted higher levels of parental stress at Wave II, controlling for covariates. Child internalising behaviour at Wave I did not predict parental stress or depression, controlling for covariates. Neither child externalising nor internalising behaviour predicted parental relationship conflict. The overall findings demonstrate that child behaviour likely influenced parental stress during the Covid-19 pandemic. Findings suggest that mental health interventions for children and parents may improve the family system during times of disaster.

## Background

### Introduction

Coronavirus (Covid-19), also known as severe acute respiratory syndrome coronavirus 2 (SARS-CoV-2), was declared a global pandemic in March of 2020 by the World Health Organization [1]. As of July 2021, there were more than 194 million reported Covid-19 cases, and more than 4 million deaths [2]. Attempting to slow down the rapid growth of this highly contagious, novel coronavirus, many countries across the globe imposed movement restrictions or lockdowns. Although these strategies were implemented to mitigate Covid-19 transmissions, these lockdowns may have had unintended negative consequences, particularly for families with children. During the pandemic many families worldwide experienced the closure of schools and childcare agencies, were forced to adapt to distance learning, faced social isolation, were unable to receive educational and social services and experienced financial strain [3]. The pandemic also fundamentally changed many families’ daily environments, in many cases restricting access to normal places of work, education and recreation. Preliminary research has shown that parents were particularly negatively impacted by the pandemic, with one study finding that 46% of parents in the United States reported high stress levels related to the pandemic compared with 28% of adults without children [4]. Caregivers also reported heightened stress and increased caregiver demands related to Covid-19 [5]. Similarly, children experienced high rates of mental health problems including anxiety, depression, sleep issues and post-traumatic stress disorder (PTSD) during the pandemic [6–8].

Relationships between family members may also have been affected by the pandemic. Researchers and practitioners have raised serious concerns about the potential impact of the pandemic on intimate partner violence (IPV; [9,10]), with some locations experiencing increases in domestic violence calls since the start of the pandemic [11,12]. Limited research has shown that parental mental health during the pandemic was linked to child–parent conflict [13]. Given the hypothesised effects of the Covid-19 pandemic on family relationships, the goal of the current study is to examine relationships between child externalising (observable aggressive, hyperactive and sometimes delinquent behaviour that is harmful to others [14]) and internalising behaviour (self-directed emotions, such as worry, fear and sadness [15]) and parental adjustment during the Covid-19 pandemic. Specifically, we examine whether child behaviour predicted parental depression, stress and inter-partner relationship conflict.

### Transactional models of parent–child behaviour

The current study is informed by transactional models of parent–child behaviour [16–18]. These models recognise that parent effects on children and child effects on parents are not independent; instead, parents and children affect each other’s behaviour bidirectionally [16–18]. Difficult child behaviour and temperament may elicit negative parental behaviour, including poor parenting and child maltreatment, which adversely influences the child’s future behaviour [19].

Although transactional models argue that parent–child effects are bidirectional, researchers have pointed out that many studies continue to assume and examine only parent-driven effects on child behaviour rather than child-driven effects on parental mental health [20–24]. Despite this, there is a growing body of research showing that child behaviour influences parental wellbeing outcomes, including family and marital conflict. Several studies have found that parents of children with adjustment issues, including infant colic and adolescent externalising problems, were more likely to consider themselves ineffective parents and have negative perceptions of their marriages [24–27]. Other studies have shown that the disruptive behaviour of infants, children and adolescents predicted long-term familial and marital conflicts [28,29]. In addition, child behaviour problems have been found to predict parental stress [22,30,31]. Child externalising and internalising behaviours are also positively associated with parental depressive and internalising symptoms [32,33]. Conversely, children’s typical development has been linked to a decrease in parental stress and depression [34,35]. Together, existing research suggests that child externalising and internalising behaviour likely impacts the quality of marital relationships, as well as parental depression and stress. However, limited research has examined the effects of child behaviour in the context of disasters, periods during which child and parent behaviour and mental health problems may be exacerbated.

### Effects of disasters on child behaviour, family functioning and parental wellbeing

Transactional effects of parent–child behaviour (also called reciprocal effects) are particularly relevant in the context of the Covid-19 pandemic given the impact of the pandemic on both child and parent adjustment. Rates of severe depression among parents during the Covid-19 pandemic were found to be over two-times higher than before the pandemic [36]. Children’s internalising and externalising behaviours have also increased compared with pre-pandemic levels [36]. Researchers have suggested that the Covid-19 pandemic has also impacted family systems, including reciprocal parent–child relationships, although this has yet to be fully examined [37]. Among the existing limited research during the Covid-19 pandemic, in a cross-sectional study of Singaporean families with children, higher parental stress was associated with harsh parenting and less parent–child closeness [34]. A longitudinal study of families within the United States found that financial difficulties were linked to decreases in parenting quality during the pandemic in families with children [36]. In a study of Japanese children, stay-at-home orders that required children to attend school remotely were associated with increases in parental stress, likely due to parents taking on more responsibilities or being unable to find childcare arrangements [38]. In a cross-sectional study of Italian families with children, higher levels of parental stress during the pandemic predicted less parental involvement with children, less concern for children’s wellbeing, and less time spent with children [39]. These studies suggest that family systems were impacted by the pandemic, although existing research has not yet fully examined the impact of child behaviour on later parental adjustment during the Covid-19 pandemic.

### Current study

The current study examines relationships between child behaviour, parental depression, parental Covid-related stress and parental relationship conflict during the Covid-19 pandemic using survey data collected between 17 April 2020 and 13 July 2020 and 17 October 2020 and 31 January 2021. We aim to address the following research question: Does child externalising and internalising behaviour predict subsequent parental depression, stress and relationship conflict? We hypothesise that parents of children with higher levels of internalising and externalising behaviour problems at baseline will experience increases in depression, stress and relationship conflict 6 months later.

## Methods

### Participants and procedures

Data were collected as part of the Covid-19: Global Social Trust and Mental Health Study [40]. This study examines the short- and longer-term effects of Covid-19 on people’s mental health, physical health and social trust in others. This study involved three online surveys (baseline, 6-month follow-up and 12-month follow-up), which were 20–30 minutes long. The first set of surveys were completed from 17 April 2020 to 13 July 2020 (Wave I), followed by surveys 6 months later at Wave II (17 October 2020–31 January 2021) and Wave III (17 April 2021–31 July 2021). Participants aged 18 years and older were recruited through convenience sampling. The study link was available in seven languages and distributed through various social media channels and personal contacts. To account for order effects, all participants completed the same questions in a random order about their living situations, relationships, mental health, and for parents, additional questions about their children’s mental health and behaviour.

**Figure 1:**
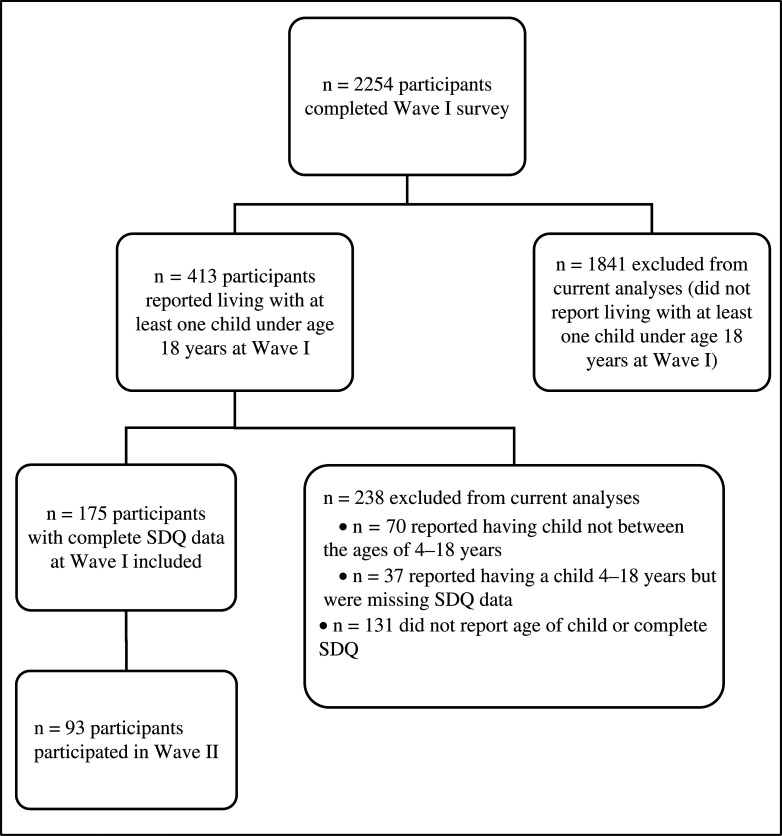
Participant flow chart.

The current analyses use data from Waves I and Waves II (data repository DOI: 10.5522/04/16583861). A total of 2254 participants from 66 countries completed the Wave I survey and 1164 participants completed the Wave II survey. Analyses for the current paper were limited to the 175 participants who reported living with one or more children under age 18 years at Wave I and who reported on children’s internalising and externalising problems using [41] the Strengths and Difficulties Questionnaire (SDQ) (see below) about at least one child ages 4–18 years. Ninety-three of these 175 participants participated in Wave II. A participant flow diagram can be found in Figure 1. At Wave I, included participants had a mean age of 43.45 years (standard deviation [SD] = 6.90) and were 80% female. Of the participants, 78.9% reported being married. A total of 83.4% of the sample had a bachelor’s degree or higher and 91.38% of the sample was either working or a student. Among these participants, 42.9% lived in the United Kingdom, 22.3% lived in Greece, 8.6% lived in the United States and the remaining 26.2% were distributed across 24 countries. A complete list of countries can be found in Appendix Table 1.

### Measures

#### Strengths and difficulties questionnaire

At Wave I, parents of children ages 4–18 years completed the SDQ [41]. The SDQ assesses positive and negative psychological attributes in children. The current study used the parent-reported questionnaire, which consists of 25 items divided into one positive attribute subscale (prosocial behaviour) and four negative attribute subscales further defined as internalising problems (emotional symptoms, peer problems) and externalising problems (conduct problems, hyperactivity/inattention). Relevant items were summed to create SDQ subscales. Parents were asked to complete the SDQ separately for each of their children (up to a maximum of five children). For the current analyses, we used data from a focal child with the highest level of total behaviour problems.

#### Patient health questionnaire

Parents completed the Patient Health Questionnaire-9 (PHQ-9; [42]) at Waves I and II. The PHQ-9 is a nine-item well-validated self-report measure of depressive symptoms over the last 2 weeks, which when summed created a total depression score.

#### Stress level

At Waves I and II, participants were asked whether they had experienced a series of 26 stressors related to the Covid-19 pandemic. For each stressor endorsed, participants were asked to report the level of stress caused by the stressor ranging from 0 (‘relaxed’) to 4 (‘a lot of stress’). Stressors that were not endorsed were given a stress level of 0. Stress levels were summed across the 26 stressors to create a total stress level score. We used the stress level score, rather than sum of the total number of stressors experienced, as individuals may vary in the extent to which they consider a particular stressor to be problematic.

#### Relationship conflict

Participants who reported being married, in a civil partnership, cohabitating or in a relationship (but not cohabitating) at Wave II completed the Marital Coping Inventory-Conflict Scale [43]. Participants were asked to think about problems with their partner in the past 6 months and report how they dealt with those problems. Participants reported on 15 items reflecting marital conflict (e.g., ‘yelled or shouted at my partner’; ‘picked fights with my partner over small issues’). Participants rated each item on a 5-point Likert scale ranging from 1 (‘Never’) to 5 (‘Usually’). This questionnaire was added to the study in Wave II and was not available in Wave I. Analyses involving relationship conflict were limited to the 163 participants who reported living with a child under age 18 years at Wave I and reported being in a relationship at Wave II.

#### Demographic covariates

We controlled for parents’ age, focal child sex (0 = male; 1 = female), and focal child age group (early childhood [4–8 years]; middle childhood [9–12 years]; and adolescence [13–18 years]; dummy coded with early childhood as the reference category) in all analyses.

### Statistical analyses

We first calculated descriptive statistics by sex and age group and performed bivariate correlations between continuous variables using IBM SPSS Statistics Version 26 (IBM Corp, Armonk, NY, USA). We used independent samples t-tests to test for sex differences in study variables and one-way analysis of variance (ANOVA) to test for differences by age group. We also used independent samples t-tests and chi (χ)-squared tests to test for differences between participants who did not participate in Wave II and those that participated in both waves. We then conducted a series of regression analyses predicting Wave II parental depression, stress levels and relationship conflict using Mplus Version 8 (Muthén & Muthén, Los Angeles, CA, USA). Child scores on the SDQ subscales were entered as predictors along with demographical covariates. We controlled for Wave I parental depression and stress (relationship conflict data was not available at Wave I) in their respective regression models in order to determine whether child behaviour predicted a change in parental mental health outcomes from Wave I to Wave II. Missing data in regression analyses were handled using full information maximum likelihood.

## Results

### Descriptive statistics and bivariate correlations

Participants who dropped out at Wave II did not differ from those that participated in both waves in terms of Wave I parental depression (t = 0.30, df = 173, *P* = 0.76), Wave I parental stress level (t = −1.78, df = 171, *P* = 0.077), Wave I child externalising behaviour (t = −0.71, df = 173, *P* = 0.48), Wave I child internalising behaviour (t = 0.11, df = 173, *P* = 0.91), or Wave I child age group (χ*^2^* = 4.06, df = 2, *P* = 0.13). Parents who participated in both waves were older at Wave I (M = 44.41 years, SD = 6.91) than those who only participated in Wave I (M = 42.35 years, SD = 6.76; t = −1.98, df = 173, *P* = 0.049). Descriptive statistics for the full sample and by child sex are shown in Table 1. Boys had higher levels of externalising behaviour than girls (t = 2.84, *P* = 0.005). All other sex differences were non-significant (*P* > 0.05). Descriptive statistics by child age group are shown in Table 2. Children in middle childhood (9–12 years) had the highest level of internalising behaviour problems (F = 4.06, *P* = 0.019). Parents of young children had the highest levels of depression at both Wave I (F = 3.51, *P* = 0.032) and Wave II (F = 3.86, *P* = 0.025).

**Table 1.** Descriptive statistics for the full sample and by child sex

	Full sample			Males			Females			t	*P*
	N	Mean	SD	N	Mean	SD	N	Mean	SD		
Wave 1 Variables											
Child externalising	175	6.14	3.69	99	6.85	3.68	74	5.27	3.54	2.84**	0.005
Child internalising	175	4.89	3.97	99	5.23	4.19	74	4.32	3.60	1.50	0.14
Parental depression	175	6.22	5.11	99	6.14	4.74	74	6.16	5.49	−0.027	0.98
Parental stress level	173	14.42	11.51	98	15.47	12.65	73	13.18	9.76	1.29	0.20
Parent age (years)	175	43.45	6.90	99	44.22	7.01	74	42.56	6.61	1.58	0.12
Wave 2 Variables											
Parental depression	81	6.09	5.59	47	6.11	5.52	33	5.55	4.99	0.47	0.64
Parental stress level	79	14.91	10.45	46	15.61	10.93	32	13.66	9.85	0.81	0.42
Marital conflict	70	28.96	9.11	41	28.59	9.01	28	29.21	9.43	−0.28	0.78

Note: Child externalising and internalising behaviours were measured using the SDQ. Parental depression was measured using the PHQ. Marital conflict was measured using the Marital Conflict Inventory. Independent samples t-tests were used to test for sex differences.

**P < 0.01.

**Table 2.** Descriptive statistics by child age group

	Young children (4–8 years)			Middle childhood (9–12 years)			Adolescents (13–18 years)			F	*P*
	N	Mean	SD	N	Mean	SD	N	Mean	SD		
Wave 1 Variables											
Child externalising	84	6.13	3.50	40	6.88	4.15	44	5.64	3.72	1.18	0.31
Child internalising	84	4.17	2.96	40	6.30	5.02	44	5.05	4.37	4.06*	0.019
Parental depression	84	7.12	5.61	40	6.30	5.07	44	4.66	3.38	3.51*	0.032
Parental stress level	84	15.86	12.36	39	13.08	10.92	43	13.53	10.69	1.01	0.37
Parent age (years)	84	39.37	4.56	40	44.59	5.89	44	49.51	6.00	54.59***	<0.001
Wave 2 Variables											
Parental depression	34	7.79	6.35	18	4.50	3.99	26	4.58	3.84	3.86*	0.025
Parental stress level	33	16.52	10.56	18	12.61	8.82	25	14.08	11.36	0.90	0.41
Marital conflict	31	29.16	10.12	16	29.75	9.40	21	27.62	7.78	0.28	0.76

Note: Child externalising and internalising behaviour were measured using the SDQ. Parental depression was measured using the PHQ. Marital conflict was measured using the Marital Conflict Inventory. One-way ANOVA was used to test for differences between age groups.

*P < 0.05. ***P < 0.001.

Bivariate correlations are shown in Table 3. Wave I child externalising behaviour was significantly associated with Wave I (r = 0.32, *P* < 0.001) and Wave II parental depression (r = 0.35, *P* = 0.001). Child externalising behaviour was also significantly associated with Wave I (r = 0.20, *P* = 0.008) and Wave II parental stress (r = 0.35, *P* = 0.002). Wave I child internalising behaviour was significantly associated with parental depression at Wave I (r = 0.27, *P* < 0.001) and Wave II (r = 0.23, *P* = 0.041). Child internalising behaviour was not associated with parental stress at Wave I (r = 0.11, *P* = 0.16) or Wave II (r = 0.18, *P* = 0.12). Wave II relationship conflict was not significantly associated with child externalising (r = 0.01, *P* = 0.95) or child internalising behaviour (r = −0.05, *P* = 0.66). Wave II relationship conflict was significantly associated with Wave I parental depression (r = 0.26, *P* = 0.029) and Wave II parental depression (r = 0.31, *P* = 0.009). Similarly, Wave II relationship conflict was associated with parental stress at Wave I (r = 0.27, *P* = 0.029) and parental stress at Wave II (r = 0.38, *P* = 0.002).

**Table 3.** Bivariate correlations

	1	2	3	4	5	6	7	8
1. Child externalising	—							
2. Child internalising	0.47*** n = 175	—						
3. Wave 1 Parental depression	0.32*** n = 175	0.27*** n = 175	—					
4. Wave 1 Parental stress level	0.20** n = 173	0.11 n = 173	0.50*** n = 173	—				
5. Parent age (years)	−0.06 n = 175	0.03 n = 175	−0.29*** n = 175	−0.18* n = 173	—			
6. Wave 2 Parental depression	0.35** n = 81	0.23* n = 81	0.59*** n = 81	0.38** n = 79	−0.19 n = 81	—		
7. Wave 2 Parental stress level	0.35** n = 79	0.18 n = 79	0.54*** n = 79	0.60** n = 78	−0.16 n = 79	0.61*** n = 79	—	
8. Wave 2 Marital conflict	0.01 n = 70	−0.05 n = 70	0.26* n = 70	0.27* n = 68	−0.02 n = 70	0.31** n = 70	0.38** n = 68	—

*Note.* Child externalising and internalising behaviour were measured using the SDQ. Parental depression was measured using the PHQ. Marital conflict was measured using the Marital Conflict Inventory.

*P < 0.05. **P < 0.01. ***P < 0.001.

### Predictors of Wave II relationship, parental depression and stress level

Results of ordinary least squares (OLS) regression analyses are shown in Table 4. Child externalising behaviour was not significantly associated with Wave II parental depression, controlling for covariates (B = 0.28, SE = 0.16, *P* = 0.08). Child internalising behaviour did not significantly predict Wave II parental depression (B = 0.12, SE = 0.13, *P* = 0.38). Child externalising behaviour (B = 0.68, SE = 0.31, *P* = 0.025), but not child internalising behaviour (B = 0.022, SE = 0.25, *P* = 0.93), significantly predicted Wave II parental stress. Neither child externalising (B = 0.13, SE = 0.39, *P* = 0.74) nor child internalising behaviour (B = −0.17, SE = 0.31, *P* = 0.58) predicted Wave II relationship conflict.

**Table 4.** OLS regression models

	Dep. Wave II Parental depression				Dep. Wave II Parental stress				Dep. Wave II Marital conflict			
	B	SE	β	*P*	B	SE	β	*P*	B	SE	β	*P*
Child externalising	0.28	0.16	0.19	0.080	**0.68**	**0.31**	**0.24**	**0.025**	0.13	0.39	0.051	0.74
Child internalising	0.12	0.13	0.082	0.38	0.022	0.25	0.009	0.93	−0.17	0.31	−0.074	0.58
Female child	−0.62	1.12	−0.056	0.58	0.55	1.97	0.027	0.79	0.58	2.36	0.032	0.81
Middle childhood	−2.43	1.47	−0.19	0.10	−0.22	2.59	−0.009	0.93	0.88	2.97	0.041	0.77
Adolescence	−2.20	1.45	−0.18	0.13	−0.11	2.70	−0.005	0.97	−1.40	3.05	−0.069	0.65
Parent age	0.066	0.087	0.08	0.45	0.02	0.17	0.013	0.91	0.026	0.20	0.020	0.90
Wave I Parental depression	**0.54**	**0.099**	**0.50**	**<0.001**	—	—	—	—	—	—	—	—
Wave I Parental stress	—	—	—	—	**0.49**	**0.08**	**0.55**	**<0.001**	—	—	—	—

Note: Child externalising and internalising behaviour were measured using the SDQ. Parental depression was measured using the PHQ. Marital conflict was measured using the Marital Conflict Inventory. Significant coefficients are highlighted in bold. Dep = Dependent variable. Missing data were handled using full information maximum likelihood.

## Discussion

The goal of the current two-timepoint study was to determine whether child behaviour during the Covid-19 pandemic predicted subsequent parental depression, stress and relationship conflict. We found that higher levels of child externalising behaviour predicted an increase in parental stress 6 months later. Contrary to expectations, child internalising behaviour did not significantly predict parental stress or depression. Neither child externalising nor internalising behaviour were associated with parental relationship conflict. Findings for externalising behaviour and parental stress are consistent with transactional models of parent–child behaviour, which argue that child behaviour influences later parental behaviour. Importantly, data from the current study were collected during the Covid-19 pandemic, a period during which parental stress and child behaviour problems may have been heightened given the major changes in the broader environment, providing unique insights into the effects of child behaviour on parental adjustment.

While the Covid-19 pandemic and subsequent lockdowns are novel, prior research has examined the effects of natural and manmade disasters on children and parents. Like Covid-19, economic recessions and natural disasters often create uncertainty and stress, especially for families. For example, research into families who experienced Hurricane Sandy in 2012 showed that pre-hurricane child depression predicted elevated post-hurricane maternal depression [44], suggesting that child psychopathology can influence parental mental health in times of disaster. Parental psychopathology has also been found to impact children during times of disaster. Children whose mothers had symptoms of PTSD and depression due to the September 11, 2001 terrorist attacks in the United States had higher behaviour problems when compared with their peers whose mothers did not experience 9/11-related psychopathology [45]. After the Boston Marathon bombings in 2013, Boston adolescents’ externalising problems increased [46], suggesting that disasters can directly impact children’s maladaptive behaviour. Families who are exposed to traumatic disaster events, like Hurricane Katrina in 2005, have reported mental health issues at a rate twice as high as families who were not disaster-exposed [47]. Financial strain can also impact disaster-exposed families by increasing parents’ feelings of ineffective parenting and depression [47,48]. Unsurprisingly, children also feel the effects of disasters and can display PTSD at higher rates than their unexposed counterparts [49]. Together with the current findings, this research highlights the importance of considering the joint effects of disasters on parents and their children.

Although these prior disasters shared characteristics of the Covid-19 pandemic in that they involved uncertainty, often had long-lasting impacts and many involved fatalities, they differed from the current pandemic in key ways. In particular, pandemic responses involve isolation and separation from others, rather than the coming together of victims which occurs in most other disaster situations [50]. Additionally, the period of uncertainty and isolation was prolonged in the current pandemic as compared to many other disasters. Limited prior research has shown that pandemic responses may have mental health effects on parents and children. One study found that children and parents in the United States who experienced isolation or quarantine during the H1N1 pandemic reported higher levels of PTSD and that children of parents who had clinically significant levels of PTSD were more likely to have clinically significant levels of PTSD themselves [50]. Along with the current findings, these results suggest that parents and children may display similar levels of negative mental health outcomes during periods of pandemic-related isolation. This is potentially consistent with a transactional model, although causal effects of parent–child behaviour are difficult to establish. Although research into prior pandemics is informative, the current Covid-19 pandemic differs from other pandemics in its global scale, widespread lockdowns and changes in daily environments, and death and illness toll. This makes research into the effects of the current pandemic on families novel and important for planning responses to future pandemics.

The results were partly consistently with transactional models of parent–child behaviour. Child externalising behaviour was associated with increases in parental stress, suggesting that child behaviour may have affected parental wellbeing, a key argument of transactional models. This research contributes to the growing body of transactional research demonstrating child effects on parental behaviour and mental health. A recent meta-analysis found that child externalising behaviour had a small, but significant relationship with later parental psychological distress, controlling for baseline parental functioning [24]. The effect size for child-driven effects on parents did not significantly differ from parent-driven effects on child externalising behaviour, illustrating the importance of considering the effects of children on their parents [24].

Contrary to our expectations, child internalising behaviour was not associated with parental covid-related stress or depression in this study. This is inconsistent with prior research which found that child internalising behaviour predicted higher levels of parental internalising behaviour, including depression [32,33]. This finding is also inconsistent with transactional models which would predict that child internalising behaviour would lead to changes in parental stress and depression. Although we cannot draw firm conclusions about the cause of this inconsistency, it is possible that in the context of the Covid-19 pandemic, child externalising behaviour problems were more stressful for parents than were internalising problems, as externalising problems may have been more observable to parents during periods of social isolation. Internalising problems may also have been viewed by parents to be more normative given the stressful and distressing nature of the pandemic. Alternatively, null findings may have been the result of the small sample size. We should note that although child internalising behaviour did not predict Wave II parental depression controlling for Wave I parental depression, child internalising behaviour was significantly associated with parental depression at Waves I and II in the bivariate analysis. It could be the case that the bivariate relationship between child internalising behaviour and parental depression in the current study was driven by shared familial, environmental or genetic influences. As many studies do not control for prior levels of parental behaviour, they may overestimate the relationship between child internalising behaviour and later parental depression.

Also contrary to our initial hypotheses, neither child externalising nor internalising behaviour were associated with parent’s relationship conflict. This could be attributable to the relationship conflict measure used in the current study, which measured general relationship conflict, but not child-rearing conflict specifically. Prior research found that marital conflict over child rearing in particular is linked to adolescent behaviour problems and marital dissatisfaction [29]. There may also be important moderators of the relationship between child behaviour and relationship conflict that were not assessed in the current study. For example, children adopting a mediator role may be associated with reductions in relationship conflict [51]. It has also been suggested that children may develop behaviour problems to distract parents from their own conflicts [52], which could explain the lack of relationship between marital conflict and child behaviour in the current study. It is also possible that parenting style could moderate this relationship. Alternatively, marital conflict could have decreased as parents adapted to children’s behaviour issues in the first months of the pandemic, although we could not test for this, as we did not measure relationship conflict during Wave I. Nonetheless, several studies have found that marital conflict is positively associated with child behaviour problems [29,51,53,54]. More research is needed in the context of the Covid-19 pandemic to better understand the null findings in the current study.

## Limitations and contributions

There are several limitations to the current study that should be noted. First, we were not able to test the full transactional model of parent–child behaviour as complete child externalising and internalising behaviour data were not collected during Wave II. Second, we could not determine whether child behaviour predicted change in relationship conflict, as relationship conflict data was added in Wave II of this study. Third, we did not examine moderators in the current analyses due to the small sample size and limited statistical power. Moderators, including child sex, age, disability and financial situation, will be important to assess in future research. Fourth, like many existing studies of families during Covid-19 [34,36,38,55], the current study relied exclusively on parent reports of both child and parent behaviour, which may result in reporter bias. Fifth, the use of a convenience sample and the small number of participants from the larger study who met the inclusion criteria for the current study limits the generalisability of findings. The statistical power for the analyses was also limited, which may have contributed to some non-significant findings. Finally, we should note that this was a relatively educated sample and consisted primarily of married parents. This may have contributed to the null findings for child internalising behaviour and relationship conflict, as families may have had access to resources to help them cope with Covid-19-related problems. Relatedly, sampling bias may occur in Covid-19 studies, with families who have Internet access being far more likely to participate [55].

These limitations should be viewed in light of several strengths of the current study. This study is one of few to examine child behaviour in relation to parental adjustment during the Covid-19 pandemic. The Covid-19 pandemic provides a unique context in which to study these relationships, as many participants in the current study were under lockdown restrictions during Wave I and parts of Wave II. In addition, we controlled for baseline levels of parental depression and stress, allowing us to determine whether child behaviour predicted changes in these parental outcomes. This was also important given that parents and children share environmental and genetic influences, leading to similarities in their behaviours [20]. As a result, it is difficult to isolate child-driven from parent-driven effects without controlling for baseline parental adjustment [24]. The current study also included a global sample, allowing us to draw broad conclusions about the effects of the pandemic on children and their parents. Data were also collected when many countries were experiencing periods of lockdown, providing unique insights into family dynamics during periods when many families experienced social isolation. The current study included both males and females across developmental ages. Importantly, this is one of the only studies to examine the way in which child behaviour impacted parental wellbeing during the Covid-19 pandemic.

The current findings could have implications for improving child and parent adjustment during future disasters, as well as policy responses during the ongoing Covid-19 pandemic. Given the relationship between child and parent outcomes, findings suggest that policymakers should seriously weigh the potential for adverse outcomes for both parents and children when considering school and childcare facility closures. Providing financial support to parents facing employment or financial difficulties should also be prioritised. It is also important to find ways for parents and children to maintain social connections during the pandemic, such as by allowing outdoor gatherings when safe. Keeping parks and natural spaces open when possible, may also help to reduce stress resulting from restrictions to families’ daily environments. Results also suggest that providing mental health and behavioural support for both children and parents may be more effective in improving mental health outcomes for parents and reducing levels of stress than providing support for parents alone. In addition, providing parents with coping strategies for dealing with child adjustment issues may help to reduce parental stress and depressive symptoms.

## Data Availability

The datasets generated during and/or analysed during the current study are available in the repository: http://www.doi.org/10.5522/04/16583861.

## Appendix A

**Appendix Table 1.** Participants’ country of residence in Wave I

Country	Frequency	Percent
Australia	2	1.1
Austria	2	1.1
Belgium	1	0.6
Cyprus	1	0.6
France	2	1.1
Germany	3	1.7
Ghana	3	1.7
Greece	39	22.3
Hong Kong	3	1.7
India	1	0.6
Indonesia	1	0.6
Italy	7	4.0
Jamaica	1	0.6
Japan	3	1.7
Malaysia	1	0.6
Malta	1	0.6
Netherlands	2	1.1
New Zealand	2	1.1
Norway	1	0.6
Philippines	2	1.1
Portugal	1	0.6
Qatar	1	0.6
Sweden	3	1.7
Switzerland	1	0.6
Trinidad and Tobago	1	0.6
United Kingdom	75	42.9
United States of America	15	8.6
Total	175	100.0
